# 

**Published:** 1803-02-01

**Authors:** 


### To CORRESPONDENTS.

*Mr.* Creaser's *Observations on Dr.* Pearson's *Examination of the Report of the Vaccine-Pock Committee of the House of Commons, concerning Dr.* Jenner's *claim for remuneration;—and The Account of the Discovery and Operation of a supposed new Medicine for Gout,—arrived too late for insertion in the present Number.*

*Communications are received from Mr.* Dennett *and Mr.* Washbourn.

